# The untwining of immunosenescence and aging

**DOI:** 10.1007/s00281-020-00824-x

**Published:** 2020-11-09

**Authors:** Weili Xu, Glenn Wong, You Yi Hwang, Anis Larbi

**Affiliations:** 1 Singapore Immunology Network (SIgN), Agency for Science Technology and Research (A*STAR), Immunos, Singapore, Singapore; 2 Department of Geriatrics, Faculty of Medicine, University of Sherbrooke, Sherbrooke, QC J1K 2R1 Canada; 3 Department of Microbiology and Immunology, Yong Loo Lin School of Medicine, National University of Singapore, Singapore, 119228 Singapore

## Abstract

From a holistic point of view, aging results from the cumulative erosion of the various systems. Among these, the immune system is interconnected to the rest as immune cells are present in all organs and recirculate through bloodstream. Immunosenescence is the term used to define the remodelling of immune changes during aging. Because immune cells—and particularly lymphocytes—can further differentiate after their maturation in response to pathogen recognition, it is therefore unclear when senescence is induced in these cells. Additionally, it is also unclear which signals triggers senescence in immune cells (i) aging per se, (ii) specific response to pathogens, (iii) underlying conditions, or (iv) inflammaging. In this review, we will cover the current knowledge and concepts linked to immunosenescence and we focus this review on lymphocytes and T cells, which represent the typical model for replicative senescence. With the evidence presented, we propose to disentangle the senescence of immune cells from chronological aging.

## Introduction

The interest in aging studies has grown with the number of elderly individuals in our societies. There is the opportunity to increase health span by better understanding the process of aging and why disease becomes more prevalent. Early studies on aging humans revealed the reduced capacity of leukocytes to produce certain cytokines or to proliferate in response to in vitro stimulation [1] and, thus, emerged the concept of immunosenescence, which is coined for the age-related immune erosion. While a significant number of studies focused on lymphocytes are particularly T cells, there are older theories that innate immunity may have a preponderant implication in the process and signs of aging [2]. Immunosenescence is often pointed to explain the reduce responsiveness to vaccination in older adults. However, not all elderly show hypo-responsiveness towards vaccination and some older adults are able to maintain a fully functional immune system during old age. This then poses an essential question: what is immunosenescence and when does it apply? In this review, we will cover the global changes observed in the major organ system with a focus on the immune system. We will cover the most updated findings in the field and propose why and how we should better redefine immunosenescence.

## Aging of the major organ system

**Fig. 1:**
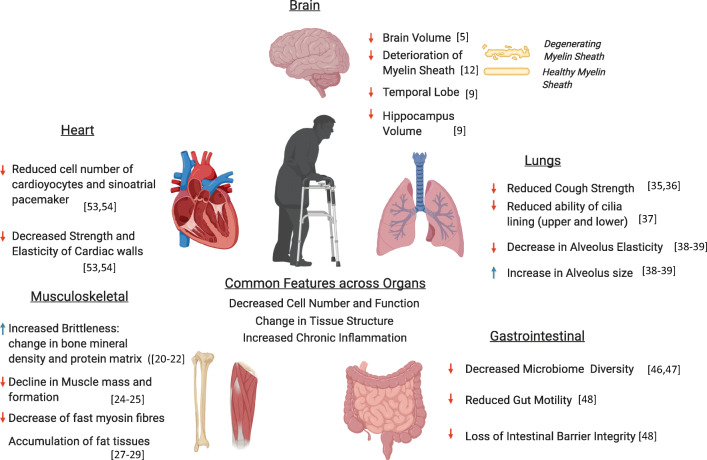
Age-related deterioration in function of various organ systems during human aging (created with BioRender.com)

Aging differentially affects the human body, with studies suggesting that different organs and even regions within the same organ can change at different rates (Fig. 1) [3]. Therefore, even if our body and the individual organ systems are of the same chronological age, we should be more focused on measuring our “biological age”, which tracks the detrimental effect of time on each of our organs, especially because these effects vary between individuals [4]. Regardless, there are some important structural and functional changes that occur in the major organs system that we will briefly cover in this section.

### Brain aging

One major organ system associated with aging is our brain and central nervous system. Aging is associated with a shrinkage in brain volume, although the exact cause of this decrease is not definitively known [5]. The prevailing theories for this observation are neuronal cell death and/or a reduction in neuronal volume [6]. In addition, changes in the neuronal structure have also been described, such as synaptic pruning [6], dendritic sprouting [7], and deterioration of the myelin sheath, which can then affect cognition [8]. Different areas of the brain exhibit different rates of shrinkage at different ages [9]. A longitudinal study found that the cross-sectional whole brain, temporal lobe, and hippocampus volumes decreased with age, with an accelerated atrophy rate after 70 years of age [9]. Multiple cross-sectional studies were also reviewed, and the authors concluded that the prefrontal cortex was the most affected area of the brain with age [10]. In addition to a decreased brain volume, aging also brings about cognitive decline and pathological diseases. Aging adversely affects the brain vasculature, and the altered blood-brain barrier and reduced cerebral blood flow are associated with white matter lesions, and correspondingly with cognitive decline [11–13]. Hypertension and altered small and large cerebro-vasculature are also associated with stroke and Alzheimer’s disease [12–14]. From an immunological perspective, the aged brain experiences an increase in an inflammatory phenotype. One key cell population proposed to contribute to the inflammation in the brain is the microglia. Microglia is an innate immune cell of the central nervous system, and in aged mouse studies, is primed for increased activation and expression of increased markers of inflammation. Following the theory that increased inflammation in the brain contributes to cognitive decline, cytokines such as type 1 IFN have been shown to negatively impact brain function [15] while IL-4 is pro-cognitive in mice [16]. In human studies, peripheral blood CD28− CD4 T cells has been shown to expand in Alzheimer’s patients compared with healthy control, contributing to low-grade inflammation [17]. Thus, an increased pro-inflammatory phenotype in the central nervous system could be associated with cognitive impairment, which can exacerbate any cognitive degeneration due to age-associated structural changes [18].

### Musculoskeletal aging

Another system often associated with aging is the musculoskeletal system. Aging brings about a deterioration of the structural integrity of the supporting skeletal structure as well as the muscles that determine our mobility, strength, and frailty. Bones are complex structures consisting of a collection of minerals, organic matrix, vasculature, and cells that grow and change in composition over one’s lifetime [19]. There is an increased risk of fracture with age caused by multiple factors such as increased brittleness due to changes in bone mineral density [20] and protein matrix [21, 22], structural weakening due to morphological changes [23], and an imbalance in bone formation, resorption, and bone repair [19].

Muscles work together with the skeletal structure to facilitate motion and mobility. Sarcopenia is defined as the decline in muscle mass and function and is one of the most significant changes caused by age that influences frailty, which is a syndrome as described by Fried and colleagues that collectively includes weakness, weight loss, low physical activity, exhaustion, and slowness [24, 25]. This decrease in muscle strength and function is due to quantitative and qualitative changes in the muscle fibres and motor unit [26]. At the muscle level, there is a loss in overall muscle fibres due to an imbalance of muscle regrowth and protein synthesis, although the exact mechanism for this decline is still under investigation and is influenced by both biological and lifestyle factors [26]. There is also a particular decrease of fast myosin isoform-expressing fibres compared with slow myosin isoform-expressing fibres, thus possibly decreasing the maximum force generated [27]. In addition, the lost fibres can be replaced by non-contractile connective or fat tissue, thus reducing the overall muscle strength even though cross-sectional area of the muscle is unchanged [28, 29]. Apart from the muscle itself, studies have implicated that loss of muscle function is linked to the age-related changes and loss of motor neurons in the motor unit. With aging, motor neurons start experiencing irregularities that adversely affect their ability to transmit signals and to restore synapses to muscle fibres after injury. Ultimately, these motor neurons, especially the largest and fastest conducting ones, are lost due to cell death [26]. One leading theory for this loss is the accumulation of DNA damage and modifications as a possible result of reactive oxygen species (ROS) generation [30]. Thus, both muscle strength and fine muscle control decline with age.

**Fig. 2:**
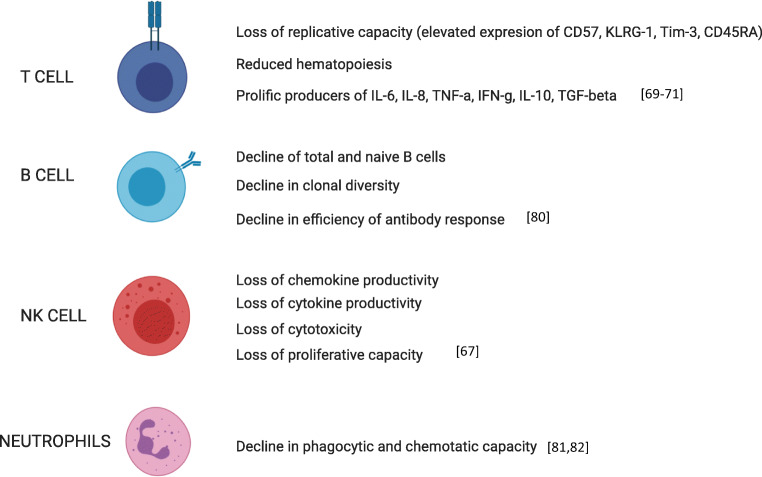
Age-related deterioration in function of various immune cell types (created with BioRender.com)

The immune system is also associated directly and indirectly with the age-associated muscle decline. Multiple immune cells (macrophages, eosinophils, and Tregs) have been implicated in appropriate muscle repair and regeneration (Fig. 2). Of special note are Tregs as they are important in the repair of injured muscle by controlling the local inflammatory responses and promoting muscle growth by releasing growth factors. There is a reduction of Treg accumulation in injured muscles of aged mice, with an associated reduction of muscle repair. This observation can be reversed by treatment with IL-33 which induces an increased Treg population and an associated enhancement of muscle repair [31, 32]. Other immune system related cytokines, such as C-reactive protein, IL-6, and TNF-α, are associated with sarcopenia which is unsurprising as they have been implicated in protein synthesis and proteolysis. As it turns out, these cytokines also tend to be upregulated in the elderly [33].

Thankfully, this age-related decline of muscle function can be ameliorated to some extent with interventions such as exercise and caloric restriction [26, 34]. Reduced muscle function also contributes to one aging-related change in the next major organ that we shall discuss.

### Lung aging

**Fig. 3:**
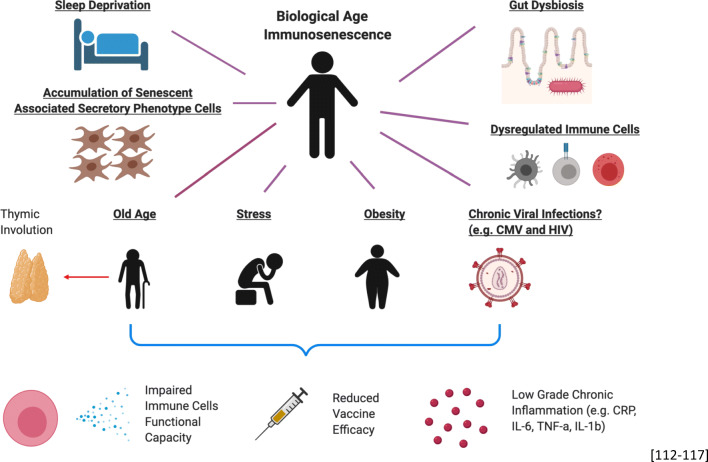
Various external stressors that results in biological age-related immunosenescence (created with BioRender.com)

The lungs are also associated with pathology in aging. As one of the organ systems constantly exposed to external stresses and allergens, the lungs play an important function as a physical as well as immunological barrier to the environment (Fig. 3). As a consequence of age-associated muscular degeneration, respiratory muscle strength is affected, leading to difficulties in breathing and reduced cough strength [35, 36]. The inability to adequately expel air, fluids, and particles from the airways is particularly detrimental during infection and disease. This is compounded by the reduced ability of the cilia lining the upper and lower airway to efficiently work to move foreign particles and mucus up and out of the airways [37]. Structurally, there is an increase in average alveolus size and decrease in alveolus elasticity, and this affects the efficiency of air exchange and susceptibility for alveolar damage during infections [38, 39].

As the lungs are one of the external facing organs, this also brings us to another important factor: the effect of aging on the immune system. After exposure to environmental allergens or infections, the immune system has to mount a delicately balanced response to the challenge to avoid lasting damage from the allergen/infectious agent or from the body’s own immune response. The immune response in aged organisms is often inappropriate, such as the innate cell response of increased pulmonary neutrophilia in the lung after exposure to cigarette smoke and increasing the risk of chronic obstructive pulmonary diseases (COPD) [40]. In the context of influenza infection, this increase in pulmonary neutrophilia is contributed by a combination of excessive neutrophil recruitment by aged alveolar epithelial cells (AECs) and an impairment of alveolar macrophage phagocytosis of apoptotic neutrophils as demonstrated by the Goldstein group in an aged murine model [41, 42]. In addition to this innate response dysfunction, the adaptive immune system also undergoes significant age-related changes. The decreased capacity for dendritic cells (DCs) to properly home and present antigens to B and T cells [43], along with the decreased naïve T cell compartment and B cell antibody-secretion capability [44, 45], mean that the adaptive immune system is less able to efficiently respond to novel infections in aged people. These inappropriate immune responses can then contribute to excessive tissue damage, reduced tissue repair, or ineffective infection clearance and therefore lead to reduced lung function [35]. Further discussion on the effects of aging on the immune system will be discussed in more details later in this review.

### Gastrointestinal aging

The gastrointestinal system is another major system that has an extensive interaction with the external environment. Of all the systems mentioned thus far, it is the most complex to study because its health and aged physiology is intrinsically linked to the microbiome contained within. There is a general consensus that with age, the microbiome changes, with community-dwelling elderly exhibiting more discrete and nursing home elderly exhibiting more drastic changes. Nonetheless, with age, the gut microbiome exhibits decreased microbial diversity as well as pathobiont overgrowth throughout the gastrointestinal tract [46, 47]. The changes in the microbiome have extensive physiological and biological effects on the gut environment as well as on the whole organism [47]. Indeed, there are many various gastrointestinal dysfunction and disorders associated with old age such as loss of intestinal barrier integrity, reduced gut motility, colitis, ulcers, cancers, and internal haemorrhage [48]. Of particular note for this review is how gut dysbiosis can trigger inflammatory responses in an organism. One study demonstrated how a microbiome transplant from aged to young mice caused local and systemic inflammatory responses in the recipients, suggesting that gut dysbiosis can drive chronic inflammation and lead to an inflammaging phenotype [49].

However, studies on the human gut have to take into account whether their observations are intrinsically linked to age, to changes in the microbiota, or both. Many of the disorders mentioned above are associated with old age but are not definitely linked to the process of aging per se. In addition, there are many other studies that observed conflicting results; for example, some animal studies reported a decline in myenteric neurons with age [50], while others reported no significant changes [51, 52]. Whether this is also observed in humans has yet to be proven. This is especially true given that human aging is influenced by the complex interplay of genetic, environmental, societal, and infection factors, thus making studying aging in the gut a particularly difficult challenge. However, if it is true that some gastrointestinal disorders are caused by changes in the microbial milieu and not by age, then careful application of prebiotics and probiotics may prove to be effective prophylactic or therapeutic treatments [47]. This may then reduce the incidence of inflammaging and age-related diseases and could be exploited as a method of ameliorating the “aging” phenotype observed in the gut.

### Cardiovascular aging

The last system that we will briefly discuss is the cardiovascular system. Much like the central nervous system, it links all the other organ systems together, transporting blood and metabolites, and can thus affect all other organ systems. In addition, blood is an easy tissue to acquire and analyse as a proxy for the overall health of the organism. The heart is at the literal heart of the cardiovascular system, and cardiac aging is associated with a change in cardiac structure. As with many other organ systems, aging leads to cell loss, in this case of cardiomyocytes and sinoatrial pacemaker cells [53, 54]. This loss corresponds with deposition of extracellular matrix, causing fibrosis and a decrease in the strength and elasticity of the cardiac walls. This change drives further adaptive remodelling of the heart structure, causing ventricular and arterial hypertrophies [53]. Together, these remodelling changes drastically modify the proper muscular and electrical functions of the heart, increasing the risks of arrhythmia and heart failure. This extracellular matrix remodelling is also observed in the vascular periphery, causing arterial stiffening and endothelial dysfunction, which increases the risk of hypertension, ischemia, stroke, and heart disease [55]. Like many other organs, apart from structural changes in the heart itself, aging also causes changes to the immune system in the heart; much like other organ systems, the heart also exhibits chronic inflammation. CD4 T cell and macrophages have been suggested as key players involved in inflammation in the heart tissue. One study showed that in aged mice, mediastinal lymph node CD4 T cells mediated increased inflammatory signals in the heart as they exhibited a stronger type 1 immune response as well as increased c-met-mediated homing to the heart tissue [56]. Macrophages have also been implicated in contributing to the inflammatory phenotype as there is an increased number of macrophages as well as macrophage-associated pro-inflammatory cytokines such as MMP-9 and CCL2 in the heart tissue [57].

With this brief overview of key structural and functional changes in multiple organ systems, one striking commonality is a decrease in cell number and cell quality, a gradual change in tissue organisation within each organ system over time, and an increase in chronic inflammation. One important general mechanism proposed for aging is the downregulation of autophagy and mitophagy in aged individuals [58, 59]. This allows damaged cells, which could display a senescence associated secretory phenotype (SASP) to persist, accumulating more DNA and cellular damage, ROS, and oxidative stress, but remains uncleared from the organism; SASP induces chronic inflammation, which has a widespread deleterious effect that is linked to the aging phenotype observed in multiple organ systems [60]. However, the mechanism behind the decreased autophagy in the elderly is yet to be fully elucidated.

## Senescence of the immune system

The term immunosenescence is now often used to describe collective changes that are observed within the immune system with age. This term “immunosenescence” has gained a negative reputation in the literature over the past decades due to its association with an increased susceptibility towards to infections, cancer, dementia, cardiovascular diseases, hypertension, diabetes, and autoimmune diseases [61–66]. Alterations in the age-dependent behaviour of T cells, natural killer (NK) cells, B cells, monocytes, and neutrophils have all been implicated in the characterization of immunosenescence [67]. While associated with chronological age, the development of immunosenescence in individuals is sensitive to environmental cues, such as immunological and infection history; it is therefore necessary to consider both influences to appreciate the diverse manifestations of immunosenescence that is observed between individuals of the same age [68].

Among different immune cell types, T cell in aging has been most meticulously characterized and includes the loss of proliferative capacity with progressive replication cycles that accelerate telomeric erosion and the accumulation of DNA damage. Senescent T cells, defined by their loss of replicative capacity, display the downregulated expression of CD27 and CD28 and can be distinguished by the elevated expression of CD57, KLRG-1, Tim-3, and CD45RA [69]. Senescent T cells are prolific producers of IL-6, IL-8, TNF, IFNγ, IL-10, and TGF-β but unable to proliferate effectively when stimulated [70]. In addition to these intrinsic properties, T cell senescence can also be described at the systemic level—this is the result of a progressive loss of lymphocyte renewal capacity through thymic involution or reduced hematopoiesis with age [71]. The latter mechanisms impede naïve cell renewal and contribute to a T cell compartment where fewer clones of late-differentiated T cells predominate [72]. Besides the accumulation of senescent T cells, naïve T cells defined by the expression of CD27 and CD45RA have been shown to be dysfunctional with age [73, 74]. However, this could be due to the purity of naïve T cells being isolated in those studies as recent studies have demonstrated that naïve T cells remain functional and it is the virtual memory (in mice), human memory T cells with naïve phenotype, and T memory stem cells that are dysfunctional during aging [75–79]. With respect to B cells, an age-associated decline in clonal diversity, the efficiency of the antibody response, and the frequencies of naïve and total B cells in peripheral blood have also been described [80].

While the study of immunosenescence has conventionally focused on lymphocyte behaviour, the expanding literature on age-associated changes in innate immune cells has allowed us to extend the usage of this term to describe functional adaptations in macrophages, neutrophils, dendritic cells, and NK cells with age [67]. For example, macrophages and neutrophils from the elderly exhibit diminished capacities for phagocytosis and chemotaxis [81, 82]. In aged mice, macrophages were more polarized towards an M2 anti-inflammatory phenotype that biased IL-10 versus IL-12 and TNF-α secretion [83]. With age, NK cells display a loss of proliferative capacity, cytotoxicity, as well as cytokine and chemokine productivity [84].

Given this perception of an age-dependent loss of function across multiple immune cell subtypes, it can be difficult to perceive immunosenescence as an adaptation rather than a consequence of aging. However, these age-associated mechanisms may have co-evolved to minimize disruption to immunological function that may accompany aging. For example, inflammaging—a systemic state of chronic low-grade inflammation that becomes more apparent with age—may reduce the threshold required for the activation of immune cells that is required for competent immunosurveillance. The observation of a heightened risk of infections in novel therapies that neutralize inflammatory molecules, such as IL-6 and TNF-α, supports the latter hypothesis and suggests that these strategies could disrupt immune homeostasis during aging [85, 86]. Therefore, studies on the aging immune system should look towards unravelling productive interactions during immunosenescence, as a broader insight of immunosenescence is necessary to facilitate anti-aging therapies—following sections discuss how we can redefine immunosenescence for this purpose.

## Rethinking Immunosenescence

### Factors influencing immune aging

Aging has been associated with a myriad of both acute and chronic diseases. At the core of these diseases, the change in the host immune system with age could either have contributed to the cause as it is the host main defence mechanism against foreign pathogens or its functionality being impacted by these diseases and conditions. These includes chronic infections such as CMV, HIV and malaria, chronic stress and glucocorticoids, memory dysfunction, bipolar disorder, and chronic inflammation that are due to immunosenescence or accelerates it [87–95]. However, the change in the immune system with age could also be seen as an adaptation process to save resources for the host rather than it being detrimental. This is because developing competent naïve T cells has only about 1–2% success rate due to the various stringent selection processes. Therefore, biological processes such as thymic involution could be seen as advantageous to the host from an energetic or evolutionary point of view [96–98]. One of the main arguments that thymic involution is detrimental to the host is due to the reduction of naïve T cells being produced, leading to a narrower repertoire for new antigens and perhaps reduced vaccine efficacy often observed in the elderly [98], while this may have been a successful programmed process for the shorter-lived humans in the past centuries and before the extended human lifespan has revealed the probable need to reverse this adaptation.

Reduced vaccine efficacy has often been observed in the elderly ever since the development of vaccines which has saved many lives from infectious diseases [99, 100]. However, recent studies have suggested that reduced vaccine efficacy is not limited to elderly individuals. Obese individuals have been reported to have reduced vaccine efficacy or impact following secondary re-challenge after vaccination even in the young [101–104]. In the elderly, studies have also shown that chronic stress, dementia, and malnutrition also have a significant impact on the efficacy of vaccination [105–108]. On the flip side, boosting of vaccine efficacy in the elderly seems possible as studies involving mTOR inhibitor such as analogs of rapamycin were able to mildly increase vaccine efficacy in the elderly [109, 110]. Metformin (a proposed drug for anti-aging) has also been included in a clinical trial to assess its potential to boost vaccine efficacy in the elderly [111]. Collectively, these studies indicate that other physiological factors are also important considerations in order for a successful vaccination to occur besides the range of T cell repertoire in the host.

### The contribution of inflammaging

Chronic low-grade inflammation is a commonality between individuals that exhibit chronic stress, obesity, aging, sleep loss, gut dysbiosis, CMV infection, dysregulated immune cell functions, and accumulation of SASP cells such as fibroblasts [112–117]. Studies have shown that chronic stress is able to induce increased levels of CRP, IL-6, TNF-α, and IL-1b [118, 119]. This is very much similar to obesity, in particular visceral fats and aging [120–126]. Chronic low-grade inflammation is defined as a higher baseline of pro-inflammatory cytokines in the circulation though the source and specific cytokines might differ slightly between these “diseases” in the absence of foreign pathogen infection. In terms of impaired immunity, both human and animal studies have shown that chronic stress reduces various immune functional capacities such as antibody production, virus-specific T cell and NK cell activities, and also the proliferation of leukocytes [127–130].

As for obesity that is also highly associated with other metabolic syndromes such as diabetes, it has been shown that insulin resistance may lead to insufficient T cell activation and also the resolution of inflammation post infection due to lack of TH2 differentiation. Besides insulin resistance, adipokines such as leptin and adiponectin are also seen to be reduced in individuals with obesity and metabolic syndromes and both play a role in initiating immune responses and resolution of inflammation respectively [131]. In response to infection, obese individuals have been reported to have a worse outcome of infection to 2009 influenza A H1N1 pandemic strain and circulating mononuclear cells exhibits a more pro-inflammatory state compared with healthy individuals [132].

In aging studies, for T cells, highly differentiated T cells, which have lower proliferation capacity, have been shown to accumulate in high frequencies and reduced productions of IL-2 in naïve T cells are some of the examples that render the T cell compartment dysfunctional in elderly individuals. As for the innate immunity compartment, macrophage phagocytic functions are reduced; neutrophil chemotaxis ability in response to infection and CD16 expression are reduced [87].

Collectively, these highlight that the presence of impaired immunity albeit having slight differences in the different scenarios and low-grade chronic inflammation could be the underlying factors that exacerbate pathology in various disease contexts.

### Immunosenescence is not age-dependent

Thus, it is important that we redefine and stress that the definition of immunosenescence is the dysfunctionality of the immune system and should encompass some features of low-grade chronic inflammation. Though this phenomenon is often seen in aged individuals, it is also possible in younger adults as it could be “accelerated immunosenescence”, especially for T cells, as shown in CMV and HIV seropositive young patients [72, 133]. Even early in life, the impact of CMV can be observed. The study from Miles et al. showed the rapid and sustained switch of the naïve/memory T cell ratio in 1-year-old infants seropositive for CMV compared with age-matched seronegative infants. Up to one-third of the pool of CD8+ T cells could exhibit loss of CD28 and CD27, which represents the profile of terminal effector T cells, many of which are in a replicative senescence stage [134]. This highlights that other factors other than chronological age could determine this level of senescence of the immune system, especially for T cells which are prone to proliferation. Looking at the other extreme, a recent study analysed the individual-level changes in the immune system profile over a 9-year period. Despite the inter-individual variability in the rates of change of some of the immune cell proportions, the authors defined an immune aging (IMM-AGE) that describes the immune status better than chronological age. This IMM-AGE score could predict all-cause mortality and could be associated to individuals with cardiovascular diseases [135]. While some aspects of immunosenescence could be associated to aging, evidence strongly suggest that responses to stressors (infectious or not) are strong modulators of this process. The concept of “accelerated biological aging” is also shown in two studies that compared biological age and chronological age in an individual, and they were able to show that individuals that have older “biological age”, as compared with chronological age, exhibit cognitive decline, looked older, self-reported worse health, and measuring lifespan (mortality) and health span (frailty) [136, 137]. Whether this is affected by the overall immunological history—the number of pathogen one experience during lifespan—is a plausible hypothesis that warrants further work. Overall, rethinking the causing agents and implications of immunosenescence will help shift the perspective that this phenomenon is not attributed to age alone, especially with the global rising rate of obesity and chronic stress of modern-day life in the young [138–141].

## Immune cell function: differentiation, adaptation, or senescence?

### Post-maturation differentiation

The immune system consists of many types of different immune cells that can be generally classified into innate and adaptive. While the immune cells in the innate immune systems generally are differentiated into its respective cell populations before exiting the bone marrow, the immune cells in the adaptive immune systems such as the classical B and T cells will require further immunological processes such as antigen presentation and undergo differentiation before being able to exert its effector functions. The differentiation stages of T cells can be identified using surface markers such as CD27 and CD45RA (or CD45RO, inversely expressed) into naïve (CD27+ CD45RA+), central memory (CD27+ CD45RA−), effector memory (CD27− CD45RA−), and terminal effector (CD27− CD45RA+), and each subset has its own define functional role in the immune system. Naïve T cells are able to proliferate effectively but not able to produce cytokines such as IFNγ while central memory T cells are able to secrete IFNγ but not effector molecules such as TNF-α. Effector memory T cells are able to secrete effector molecules, and terminal effector T cells are able to secrete a wider range of cytokines but have limited proliferative capacity. [142].

B cell differentiation stages, on the other hand, can be identified using surface markers such as CD27 and IgD into naïve (CD27− IgD+), memory B cells (CD27+ IgD+), switched memory (CD27+ IgD−), and double negative (CD27− IgD−) [143]. In general, the various immune cells undergo a linear differentiation process to achieve its various effector functions. However, there is also a certain level of plasticity for certain cell types such as ILCs and macrophages due to the environmental milieu that could alter its function, which should also be viewed as adaption [144, 145].

### Post-differentiation adaptation

Adaptation by definition is a process, whereby the subject changes certain aspects of itself to better survive in the current environment. Thus, the end goal of the process would be an evolutionary stronger organism being emerged after the current situation but only for that particular situation [146]. With this, we can view that a functional immune system should be a system that is adaptable throughout the host lifetime as its role is to protect the host from various foreign pathogens during that timeframe. Various adaptation processes of the immune system when encountered with foreign pathogens can be seen in the different immune cells ranging from innate immune cells (e.g. monocytes and natural killer cells) to adaptive immune cells (e.g. T cells and B cells).

The term innate describes the ability to perform a function by an organism as naturally inherited and not due to improvement based on the current situation which will otherwise be termed as adaptive. Thus, innate immune cells are termed as such because its effector functions do not require additional process for it to be functional in times of challenge and its magnitude of the effector function following secondary challenge does not differ as they do not possess immunological memory. However, recent studies with regard to trained immunity on monocytes and “memory” natural killer cells suggest that they might possess a certain level of adaptation or immunological memory though it could be short-lived as compared with the classical T and B cells which are long-lived. “Trained” monocytes have been shown to secrete higher level of pro-inflammatory cytokines following a second challenge by a different stimulant. After the first challenge, genes associated with pro-inflammatory cytokines are marked with H3K4m1 and H3K27ac, a signature of an open chromatin, allowing these genes to be more transcribed easily [147–149]. Even though this process has been shown to only last for up to 3 months, trained immunity can be seen as an adaptation process by the monocytes as it primes itself to react stronger to another challenge by a similar or different stimulus [150]. This process could therefore be seen as essential as the immune system could deem the current environment as dangerous that threatens the survival of the host.

Natural killer cells have been traditionally categorized as innate in the lymphoid compartment as they do not under RAG recombination and possess immunological memory [151]. However, recent studies in have shown that NKs could have a “bystander effect”, similar to the concept of trained immunity, whereby it has enhanced responses for if they were exposed to IL-12 and IL-18 prior to the second challenge in mice and “immunological memory” that leads to clonal expansion upon antigenic challenge [152–154]. These two processes thus illustrate how NKs could help the immune system to adapt to the current environment that could then enhance the survival rate of the host.

### Adaptation of differentiated immune cells

For adaptive immune cells such as T cells and B cells, differentiation is a required process for it to exert its function during foreign pathogen infections. This is observed when a naïve T cell or memory T cell differentiates into an effector cell and clonally expand during such situation. Thus, differentiation could also be seen as an adaption process by the adaptive immune system to combat the current infection. Following resolution of the infection, some of the remaining T cells differentiates into central memory, effector memory, and also tissue-resident memory T cells [155], while B cells differentiate into memory B cells and secrete antibodies in the circulation. This post-infection differentiation process is part of the adaption that allows these immune cells to respond more efficiently when challenged with the same pathogen. Altogether, this makes the organism “fitter” in the local environment, whereby the host is expected to face the same pathogen again [156].

### Senescence: the end road of adaptation

However, with the constant adaptation that is needed to ensure that the host survive, there is perhaps a limit to the adaptation process as resources could be limited. This might result in some immune cells exhibiting a replicative senescence phenotype, whereby they lose their proliferation capacity. The accumulation of such replicative senescent T and NK cells is often observed in individuals with chronic viral infections such as CMV, HIV, and also elderly individuals [134, 157–160]. This phenomenon of replicative senescent was first demonstrated by Hayflick and is also known as the Hayflick Limit, whereby the cells could not replicate due to the shortening of its telomere with each cell replication [161]. For most cell types, when they become senescent, there is a maintenance system that they are either removed by immune cells or undergo apoptosis and the functionality of this system is essential to prevent impaired immunity [162, 163]. However, this is not the same for T cells, as senescent CD8 T cells are capable to acquire a broad-spectrum, innate-like killing activity through NKG2D against viral infected cells and tumour [164, 165], suggesting that it could be a benefit to keep senescent T cells in the circulation considering that thymic involution occurs which results in the reduction of producing new competent naïve T cells. Therefore, this process could then be seen as an adaptation even though not optimal to combat past infections faced by the host in order to prevent re-activation of chronic infection.

## Conclusion

The immune system is required to go through a continuous adaptive process throughout the host lifetime, constantly changing and remodeling to ensure the host survival in an environment littered with foreign pathogens. Differentiation, trained immunity, immunological memory, and perhaps senescence are some of the mechanisms it utilizes for this adaption process to balance the use of limited resources and the survival of the host. When studying immunity in the context of aging, it is important to differentiate these processes to better translate the findings into effective measure for improving immune response in older adults. In the era of omics, the contribution of epigenetic studies would be important to define the actual age of cells we study [166]. An important factor related to immunogerontological studies is cellular turnover which varies significantly between cell types. Another important factor is replicative capacity. While some cell types such as T cells are able to proliferate (as part of their function), others proliferate much less (e.g. monocytes) and as such are less susceptible to replicative senescence. While we use cellular markers, especially by flow cytometry, to identify cell types and subtypes, we still do not understand the reason these markers are up- or downregulated. Further studies on the role of changed expression would unravel whether this is part of differentiation or an adaptation to specific stimuli. Two markers in particular are of interest in the context of T cells: (i) CD57, probably the best marker of replicative senescence, for which we still do not know the ligand and the intracellular interactome and signaling effect, and (ii) CD45, a phosphatase involved in T cell receptor signaling but which splicing variants (CD45RA, CD45RB, CD45RO) have not been investigated enough for their differential expression and signaling. Why a vast majority of replicative senescent T cells (CD57+) express CD45RA is not known. The complexity also resides in the fact senescence is not linked to one type of stimuli and that several signaling pathways may lead to senescence in the same cell type. Defining the commonalities in the process of senescence across various immune and non-immune cells would help answer some of these questions which could then be utilized to improve health span.
